# Shared lesion correlates of semantic and letter fluency in post-stroke aphasia

**DOI:** 10.1111/jnp.12211

**Published:** 2020-05-15

**Authors:** Melissa Thye, Jerzy P. Szaflarski, Daniel Mirman

**Affiliations:** 1 Department of Psychology, University of Edinburgh, UK; 2 Department of Neurology, University of Alabama at Birmingham, Alabama, USA

## Abstract

Lesion–symptom mapping studies have reported a temporal versus frontal dissociation between semantic and letter fluency, and mixed evidence regarding the role of white matter. Mass-univariate and multivariate lesion–symptom mapping was used to identify regions associated with semantic and letter fluency deficits in post-stroke aphasia. Multivariate LSM revealed broad networks including underlying white matter, and substantial overlap between both types of fluency, suggesting that semantic fluency and letter fluency largely rely on the same neural system. All data are available on OSF.

Verbal fluency is a critical component of speech production that is routinely used in clinical and research contexts for developmental and neurological assessment. Standard fluency assessments require generating as many items as possible within 1–2 min, either belonging to a semantic category (semantic fluency) or beginning with the same letter (letter fluency). These tasks require intact word knowledge, rapid and controlled retrieval of relevant items, and self-monitoring of previous productions (Robinson, Shallice, Bozzali, & Cipolotti, 2012), as well as differentially engaging semantic or phonological knowledge. The cognitive dissociation between these fluency measures has been supported by both exploratory and confirmatory factor analyses in which a two-factor solution differentiates semantic and phonological fluency tasks suggesting that these measures are capturing distinct cognitive processes, although shared variance between the factors was noted (Schmidt et al., 2017).

Previous lesion–symptom mapping (LSM) studies have localized semantic and letter fluency deficits to damage in shared cortical regions, including left inferior frontal gyrus and insula (Biesbroek et al., 2016) and parietal cortex (Baldo, Schwartz, Wilkins, & Dronkers, 2006) extending into supramarginal and angular gyri (Chouiter et al., 2016). Both semantic and letter fluency deficits were associated with damage to anterior white matter tracts including external capsule, superior and anterior corona radiata, and portions of the superior longitudinal fasciculus in one LSM study (Chouiter et al., 2016) and to anterior portions of the inferior fronto-occipital fasciculus, superior longitudinal fasciculus, uncinate fasciculus, frontal aslant tract and anterior thalamic radiations in another study (Li et al., 2017).

Evidence of a neural dissociation comes from research showing semantic fluency deficits after damage to left temporal regions (Baldo et al., 2006; Biesbroek et al., 2016; Chouiter et al., 2016) and letter fluency deficits more consistently seen after damage to frontal regions (Baldo et al., 2006; Baldo, Schwartz, Wilkins, & Dronkers, 2010; Biesbroek et al., 2016; Robinson et al., 2012). There is no converging evidence across studies concerning the role of white matter tracts in each fluency measure. This neural dissociation may arise because semantic fluency requires more temporal involvement for searching conceptual knowledge, whereas letter fluency relies on phonological word knowledge supported by frontal regions.

However, these studies have important methodological differences: inclusion of left and right hemisphere stroke cases, with one study having minimal left hemisphere coverage (Biesbroek et al., 2016); inconsistency in task administration (e.g., time limit, number of categories); and variations in how the LSM analyses were run, with several studies not accounting for lesion volume or applying an inappropriate multiple comparison correction. The latter issue may have particular relevance for the inconsistent role of underlying white matter tracts given the known mislocalization concern in mass-univariate LSM (Mah, Husain, Rees, & Nachev, 2014). In addition, the relative contributions of general impairments versus task-specific (semantic vs. letter) impairments may be misrepresented by the choice of statistical correction methods (Thye & Mirman, 2018).

The present study re-examined the shared and distinct neural correlates of semantic and letter fluency deficits using current best practices in reproducibility and LSM methods. These include adopting a reproducible lesion segmentation method, controlling for overall lesion volume, appropriately correcting for multiple comparisons, limiting analysis to regions with sufficient lesion involvement and running both mass-univariate LSM and a multivariate alternative: sparse canonical correlation analysis (SCCAN), to better capture the distributed fluency network (Pustina, Avants, Faseyitan, Medaglia, & Coslett, 2018). The data as well as all analysis code and supplemental materials are available on OSF.

## Methods

### Participants

Prospectively collected MRI and psycholinguistic data from 55 participants with aphasia secondary to a single left hemisphere stroke were analysed. All participants were previously included in other studies. Fluency was assessed with the Semantic Fluency Test and the Controlled Oral Word Association Test. Participants were given 1 min to generate as many items as possible belonging to a probed semantic category (animals, fruits and vegetables, things that are hot) or beginning with the prompted letter (C, F, L). Participant demographic information is presented in Table 1, and the lesion overlap map is available on OSF (https://osf.io/crv4f/).

### Analysis

Automated lesion segmentation was completed using LINDA (Pustina et al., 2016). After segmentation, the resulting lesion files were visually examined, and reproducible modifications were made to all lesion masks to account for consistent errors in segmentation (e.g., identifying distal clusters of healthy tissue or portions of the cerebellum as part of the lesion territory). Details of this procedure and the scripts used to modify the lesion masks are provided on our OSF page (https://osf.io/crv4f/). The OSF also has the lesion masks and behavioural scores that were used in the LSM analyses.

Mass-univariate VLSM and multivariate SCCAN were run separately for each fluency measure using the sum of the responses across the three runs of each fluency task. In order to further assess distinct recruitment of posterior temporal regions in semantic fluency after controlling for non-semantic demands, a separate analysis was run using the residuals from a model of semantic fluency accounting for letter fluency. For comparison with SCCAN, support vector regression (SVR) LSM was run as a *post-hoc* multivariate LSM analysis using the same semantic and letter fluency scores. Results were corrected for multiple comparisons using permutation-based continuous FWER correction with *v* = 100 (Mirman et al., 2018) for the VLSM analysis and FDR correction for the SVR-LSM analysis. For SCCAN, a sparseness parameter determines the extent of voxels generated in the result. This was separately optimized for each fluency measure using fourfold cross-validation, and the goodness of the overall LSM solution was assessed by cross-validated accuracy (CV correlation). All of the LSM analyses controlled for lesion size using total direct lesion volume control (Mirman et al., 2015). Each LSM analysis excluded voxels where at least 10% (*N* ≈ 6) of participants did not have lesions. All analyses were conducted in R using the lesymap package.

## Results

**Figure 1.**
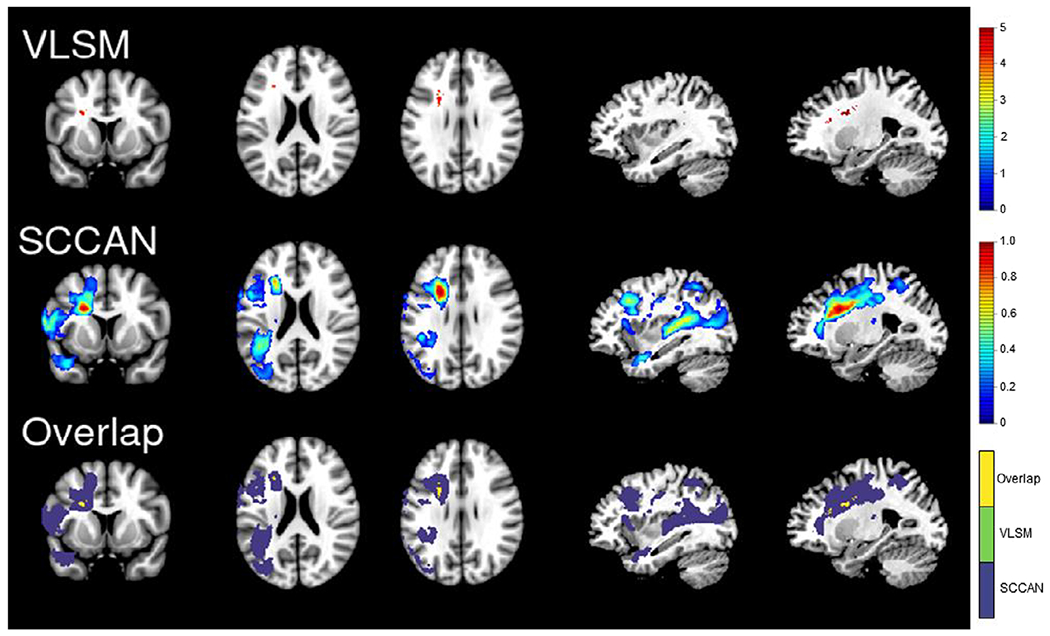
Semantic fluency results. The VLSM (top panel) and SCCAN (middle panel) results for semantic fluency. The bottom panel is showing the areas of overlap (yellow) between the VLSM (green) and SCCAN (purple) results. For ease of comparison, all results are shown on the same slices of an MNI template (from left to right: 136, 94, 101, 56 and 66). [Colour figure can be viewed at wileyonlinelibrary.com]

**Figure 2.**
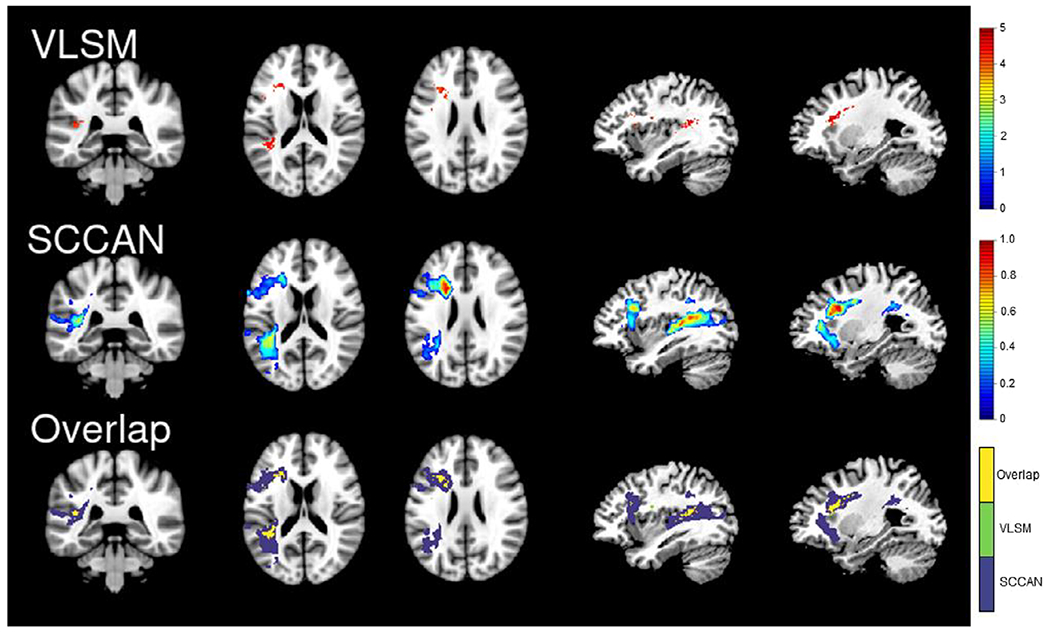
Letter fluency results. The VLSM (top panel) and SCCAN (middle panel) results for semantic fluency. The bottom panel is showing the areas of overlap (yellow) between the VLSM (green) and SCCAN (purple) results. For ease of comparison, all results are shown on the same slices of an MNI template (from left to right: 90, 91, 98, 54 and 64). [Colour figure can be viewed at wileyonlinelibrary.com]

**Figure 3.**
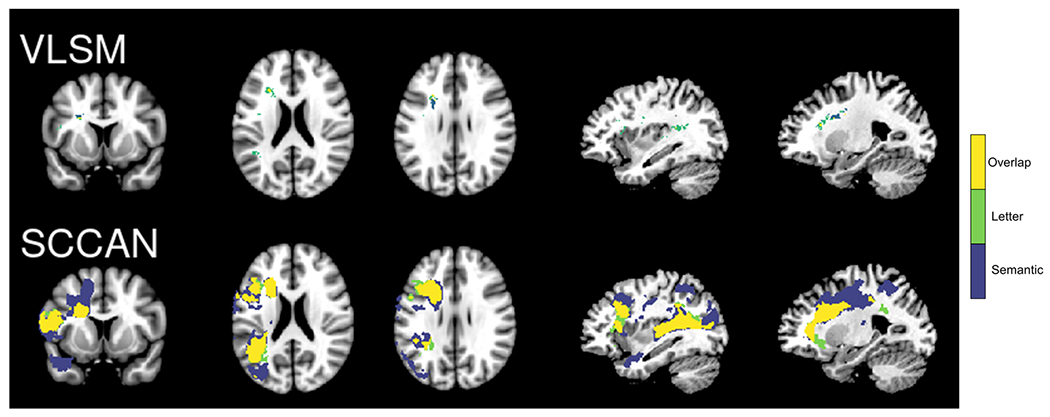
Overlap in fluency results. The areas of overlap (yellow) for the semantic (purple) and letter (green) fluency results for the VLSM (top panel) and SCCAN (bottom panel) analyses. For ease of comparison, all results are shown on the same slices of an MNI template (from left to right: 136, 94, 101, 56 and 66). [Colour figure can be viewed at wileyonlinelibrary.com]

Figures 1 and 2 show the results of the LSM analyses for semantic and letter fluency, respectively. The overlap between the semantic and letter fluency results is shown in Figure 3, and a table showing the results by region is available at https://osf.io/crv4f/.

For semantic fluency, the VLSM analysis localized the lesion–symptom association to the anterior white matter tracts, with the largest cluster located in the anterior corona radiata and smaller clusters in the external capsule and anterior limb of the internal capsule. The SCCAN results included these white matter tracts, as well as portions of the superior longitudinal fasciculus and posterior thalamic radiation. In addition, SCCAN identified a broad network of cortical regions extending from the inferior parietal lobule into superior and middle temporal gyri and temporal pole and in frontal regions including inferior frontal and middle and superior frontal gyri (optimized sparseness = 0.83, CV correlation = .52, *p* < .001).

VLSM localized deficits in letter fluency to damage to anterior and posterior white matter tracts, with the largest clusters located in the anterior corona radiata and superior longitudinal fasciculus, and grey matter regions, including the inferior frontal gyrus, insula, and middle and superior temporal gyri. The SCCAN results included these white and grey matter regions and a broader extent of the inferior and middle frontal gyri. A posterior cluster including portions of the inferior parietal lobule and angular gyri extending anteriorly into superior and middle temporal gyri was also identified, although the extent of this cluster was smaller than the cluster identified for semantic fluency (optimized sparseness = 0.40, CV correlation = .62, *p* < .001). Performance on the semantic and letter fluency tasks was very highly correlated (*r* = .88), and there were no significant results for the residuals analysis. No clusters survived FDR correction in the SVR-LSM analysis for either fluency measure.

## Discussion

There was a high degree of overlap between regions associated with semantic and letter fluency performance and no significant regions identified in the residuals analysis, suggesting that both types of fluency largely rely on the same neural systems in this sample of participants with aphasia. These results partially converge with previous studies suggesting that fluency is broadly supported by the left inferior frontal gyrus (Biesbroek et al., 2016) and portions of the parietal lobe and middle temporal gyri (Baldo et al., 2006; Chouiter et al., 2016). The key difference is that the present results suggest that both tasks rely on very similar, large networks of left hemisphere regions. In contrast to previous studies, mass-univariate LSM identified damage to white matter tracts as the primary correlate for both deficits (particularly semantic fluency), rather than frontal or temporal cortical regions. SCCAN captured a broader network of grey and white matter regions, especially for semantic fluency.

Although frontal and temporal regions were identified for both semantic and letter fluency, there were differences in the regions involved and the extent of involvement. The semantic fluency results captured a larger network of regions, including additional temporal regions that were more posterior to the regions identified for letter fluency and a portion of the middle temporal pole as well as a frontal cluster extending posteriorly into the supplementary motor area and underlying white matter. An additional, more inferior cluster extending from the inferior frontal gyrus to the insula was captured in the letter fluency network that was not seen in the semantic fluency network, although the strongest weights for both measures were localized to the white matter medial to inferior frontal regions.

In addition to the neural overlap, the high correlation between both tasks provides converging behavioural evidence that semantic fluency and letter fluency engage a common cognitive system. Previous LSM studies of semantic and letter fluency have also reported a strong, positive association (e.g., Biesbroek et al., 2016: *r* = .64; Chouiter et al., 2016: *r* = .70;Robinson et al., 2012: *r* = .69). The strength of the association between the semantic and letter fluency scores is higher in the current study which may be one factor driving the greater degree of neural overlap. Further, the high correlation may be driven by general cognitive impairments which impact overall task performance. Schmidt et al. (2017) reported that a two-factor solution that distinguished between semantic and letter fluency was preferred to a one-factor solution, but there was common variance between the factors. Further, Schmidt et al. observed that the correlation between semantic and letter fluency was higher for the participants with aphasia compared to healthy participants. That is, the two tasks may differ in their relative reliance on semantic and phonological/orthographic processes, but the shared lexical, memory, and cognitive control processes appear to be much more important, at least for performance of people with aphasia following left hemisphere stroke.

This study also demonstrates key methodological issues. When mass-univariate LSM and even SVR-LSM are implemented with lesion volume control and multiple comparison correction, the results tend to be highly focal and restricted to white matter, but this can result from statistical factors rather than unique contributions of particular white matter tracts (for additional discussion see Thye & Mirman, 2018). Previous inconsistent reports of the involvement of white matter in either semantic or letter fluency may have been driven by methodological limitations that exacerbated the mislocalization issue to which both mass-univariate and SVR-LSM are susceptible (Mah et al., 2014; Sperber, Wiesen, & Karnath, 2019). In the present study, the mass-univariate results, although overly focal, capture portions of the white matter tracts that were also given the strongest weights in the SCCAN analysis. Multivariate LSM more effectively captures the distributed network that supports fluency, rather than localizing this complex process to an artifactually focal region that survives correction. This is also among the first LSM studies to use the LINDA automated lesion segmentation algorithm and the first to share the quality control procedure (https://osf.io/crv4f/), which is an important step towards making LSM more accessible and reproducible. In addition, the lesion masks and behavioural data that were used to generate the LSM results are shared on OSF, allowing direct replication of the results reported here.

Previous LSM studies of the behavioural and neural dissociation between semantic and letter fluency report mixed findings. The present study investigated the shared and distinct neural correlates of semantic and letter fluency in post-stroke aphasia using both mass-univariate and multivariate LSM methods. Mass-univariate LSM results were overly focal, whereas the multivariate SCCAN results captured an overlapping network that included frontal and temporal regions for both fluency measures, suggesting that semantic fluency and letter fluency rely on the same broad network of regions and engage shared cognitive processes. The largely overlapping reliance of both fluency tasks on a broad language network is relevant for the use of these tasks in clinical and developmental assessment of language production.

## Figures and Tables

**Table 1.** Participant demographic information

	Mean (*SD*)	Range
Age (years)	52.82 (15.66)	22.65–90.76
Time since stroke (months)	39.80 (37.88)	2.24–167.93
Lesion volume (cc)	113 (69)	2–251
SFT	18.07 (15.63)	0–62
COWAT	8.15 (8.38)	0–36
BNT	33.18 (20.13)	0–60

	Number of participants	
Gender (M:F)	32:23	
Handedness (R:L:A)	49:4:2	

*Note*. A, ambidextrous; BNT, Boston Naming Test; cc, cubic centimetre; COWAT, Controlled Oral Word Association Test; F, female; L, left; M, male; R, right; *SD*, standard deviation of the mean; SFT, Semantic Fluency Test.

## Data Availability

The raw data are not publicly available due to privacy or ethical restrictions; however, the lesion masks and all analysis code used to run the lesion–symptom mapping analyses as well as supplemental material that support the findings of this study are openly available on Open Science Framework at https://osf.io/crv4f/.
